# Individual In-Situ GPS-Derived Acceleration-Speed Profiling: Toward Automatization and Refinement in Male Professional Rugby Union Players

**DOI:** 10.1186/s40798-023-00672-7

**Published:** 2024-01-11

**Authors:** Nathan Miguens, Franck Brocherie, Loïc Moulié, Patrick Milhet, Mathieu Bon, Pierre Lassus, Jean-François Toussaint, Adrien Sedeaud

**Affiliations:** 1 IRMES – URP 7329, Institut de Recherche Médicale Et d’Epidémiologie du Sport, Université de Paris Cité, 11 Avenue du Tremblay, 75012 Paris, France; 2 Institut National du Sport, de l’Expertise Et de La Performance (INSEP), Paris, France; 3 Laboratory Sport, Expertise and Performance (EA 7370), French Institute of Sport, Paris, France; 4 Stade Montois Rugby Pro, 270 Avenue du Stade, 40 000 Mont De Marsan, France; 5 Centre d’Investigation en Médecine du Sport, Assistance Publique - Hôpitaux de Paris, Hôtel-Dieu, Paris, France

**Keywords:** Rugby union, Testing, Sprint, Running

## Abstract

**Background:**

Recently a proof-of-concept was proposed to derive the soccer players’ individual *in-situ* acceleration-speed (*AS*) profile from global positioning system (GPS) data collected over several sessions and games. The present study aimed to propose an automatized method of individual GPS-derived *in-situ AS* profiling in a professional rugby union setting.

**Method:**

*AS* profiles of forty-nine male professional rugby union players representing 61.5 million positions, from which acceleration was derived from speed during 51 training sessions and 11 official games, were analyzed. A density-based clustering algorithm was applied to identify outlier points. Multipl*e AS* linear relationships were modeled for each player and session, generating numerous theoretical maximal acceleration (*A*_*0*_*)*, theoretical maximal running speed (*S*_*0*_*)* and *AS* slope (*AS*_slope_, *i.e.*, overall orientation of the *AS* profile). Each average provides information on the most relevant value while the standard deviation denotes the method accuracy. In order to assess the reliability of the *AS* profile within the data collection period, data were compared over two 2-week phases by the inter-class correlation coefficient. *A*_*0*_ and *S*_*0*_ between positions and type of sessions (trainings and games) were compared using ANOVA and post hoc tests when the significant threshold had been reached.

**Results:**

All *AS* individual profiles show linear trends with high coefficient of determination (r^2^ > 0.81). Good reliability (Inter-class Correlation Coefficient ranging from 0.92 to 0.72) was observed between *AS* profiles, when determined 2 weeks apart for each player. *AS* profiles depend on players’ positions, types of training and games. Training and games data highlight that highest *A*_*0*_ are obtained during games, while greatest *S*_*0*_ are attained during speed sessions.

**Conclusions:**

This study provides individual *in-situ* GPS-derived *AS* profiles with automatization capability. The method calculates an error of measurement for *A*_*0*_ and *S*_*0*_, of paramount importance in order to improve their daily use. The *AS* profile differences between training, games and playing positions open several perspectives for performance testing, training monitoring, injury prevention and return-to-sport sequences in professional rugby union, with possible transferability to other sprint-based sports.

**Key Points:**

- *AS* profiles computed from rugby union GPS data provide positional benchmarks during training and competition.
- This study provides automatic detection of atypical data and the computation of error measurement of theoretical maximal acceleration and speed components.
- This refinement constitutes a step forward for a daily use of ecological data by considering data collection and method reliabilities.
- This easy-to-implement approach may facilitate its use to the performance management process (talent identification, training monitoring and individualization, return-to-sport).

## Background

In invasion and combat sports such as rugby union, the ability to cover a distance in the shortest possible time (or the largest distance in a given time) is a key determinant (*e.g.*, for breaking the line, avoiding or tackling an opponent, scoring a try), independently of the player level or position [1–5]. Examining such acceleration capabilities through velocity–time measurements and force–velocity profiling [6–8] is of paramount importance to individualize players’ training process [6, 9, 10]. However, although being simple, valid and reliable [6, 11], actual force–velocity profiling has several technical constraints: it is a time-consuming testing method using dual-beamed photocells, radar devices, instrumented treadmill or track-embedded multiple force plate systems, that may all limit their daily use [12].

The advent of player tracking technologies such as global positioning system (GPS) and local positioning systems (LPS) allows for the relatively unobtrusive, objective and simultaneous monitoring of players’ locomotion during training and games [12–15]. Advancements in sensor technology have facilitated a transition from descriptive examinations of movement patterns to the comparative analysis of activity profiles and the establishment of competition standards [16]. This progression has also unlocked the potential for leveraging acceleration, deceleration, and high-speed data [17]. Consequently, a wide variety of metrics to assess acceleration and the ability to change velocity in training and competition have emerged [17, 18]. Morin et al. [19] recently proposed to measure individual *in-situ* acceleration-speed (*AS*) profile of soccer players computed from GPS training values. Conceptually, this easy-to-implement concept is resembling the force–velocity profile derived from single straight sprinting test [6, 7] with the maximal theoretical acceleration (*A*_*0*_) and force (*F*_*0*_) expressing the maximal acceleration/force capability in the antero-posterior direction and the maximal theoretical running speed (*S*_*0*_) being the mechanical equivalent of maximal theoretical velocity (*V*_*0*_) [19]. Although promising, such non-intrusive *AS* profiling may be applied to other team-sport datasets that may include competitive sessions in order to provide sport-specific standards [20]. Recently Clavel et al. [21] demonstrated a nearly perfect correlation between radar and GPS-derived force–velocity variables. This study indicated that the GPS device is a valid, reliable, and time-effective alternative to radar for force–velocity assessment. Furthermore, a recent study in elite female soccer players demonstrated that *AS* in-situ profiles derived from both training sessions and games align closely with isolated *AS* sprint profiles [22]. However, questions have arisen regarding the methodologies employed in profile creation specifically in terms of the analysis methods such as data cleaning, outlier removal, and signal processing [22]. Other major improvement would be to provide an error of measurement on *A*_*0*_ and *S*_*0*_ variables for each training type (*e.g.*, speed *vs*. scrimmage) and game, a major component to improve any decision-making process [23]. Furthermore, the increasing volume of data now calls for an automatized development of algorithms for fast and accurate data processing, possibly through open-source repositories.

It may also be important to define profiles according to playing position and individual idiosyncrasies [7]. In rugby union, due to variability in anthropometrics and body composition among players [24, 25] coupled with position-specific game demands [3, 26], substantial inter-player variations have been identified in acceleration and speed capacities. Notably, forward players exhibit a force-dominant profile, in contrast to the velocity-dominant profiles observed among backs [27]. Thus, a better understanding of the individual position-specific *AS* analysis should have practical implications to design personalized targeted training intervention (*e.g.*, replicating or exceeding position-specific match-play demands, acceleration/force or speed/velocity-oriented drills) accommodating for each player strengths or weaknesses [10].

The purpose of this study was to propose an automatized method to determine individual *in-situ* GPS-derived *AS* profiles and generate an error of measurement on *A*_*0*_ and *S*_*0*_ components in reference to training types, games and positions in professional male rugby union players.

## Methods

### Ethics Statement

This study was approved by the Institut de Recherche bio-Médicale et d’Epidémiologie du Sport scientific committee and registered within the Commission Nationale de l’Informatique et des Libertés (CNIL) with the following registration number: 2224815. Data collection was compliant with the General Data Protection Regulations applied in the European Union and conform to the general ethical principles of the Declaration of Helsinki.

### Data Sample

Forty-nine male rugby union players (mean ± SD; age: 27.2 ± 5.9 yr, height: 184.2 ± 8.0 cm, body mass: 99.6 ± 17.1 kg) belonging to a French second division (ProD2) squad (ten first rows, seven second rows, ten third rows, three half scrums, two half backs, three centers, seven wings, three backs) were enroled. Over a 5-months period (*i.e.*, from Febrary 5th to June 7th 2021), they carried a global positioning system (GPS) sensor (Catapult Vector X7, Firmware: 8.1.0, Melbourne, Australia) sampled at 10 Hz during training sessions and games. According to the manufacturer guidelines and methodological considerations on minimum effort duration to quantify high-intensity efforts using GPS [28], acceleration and speed were identified only when effort duration exceeded a threshold of 0.4 s. Horizontal dilution of precision over the period averaged 0.76 ± 0.06. The mean number of connected satellites was 15.48 ± 0.95. The feature for exporting raw data in CSV format within Openfield was used. Acceleration data were derived from speed data with a time interval (referred in the software as Smoothing Filter Width) of 0.2 s. Of the forty-nine players, two first rows and two centers were excluded from the study, due to long-term absence. Raw GPS data from 51 trainings (*i.e.*, 3–4 sessions per week including speed session, specific forwards or backs trainings, scrimmage session or lineout session), 11 official games (*i.e.*, one per week), and other sessions representing a dataset of 61.5 million positions, speed and accelerations, were analyzed.

### Outlier Points’ Identification

**Fig. 1:**
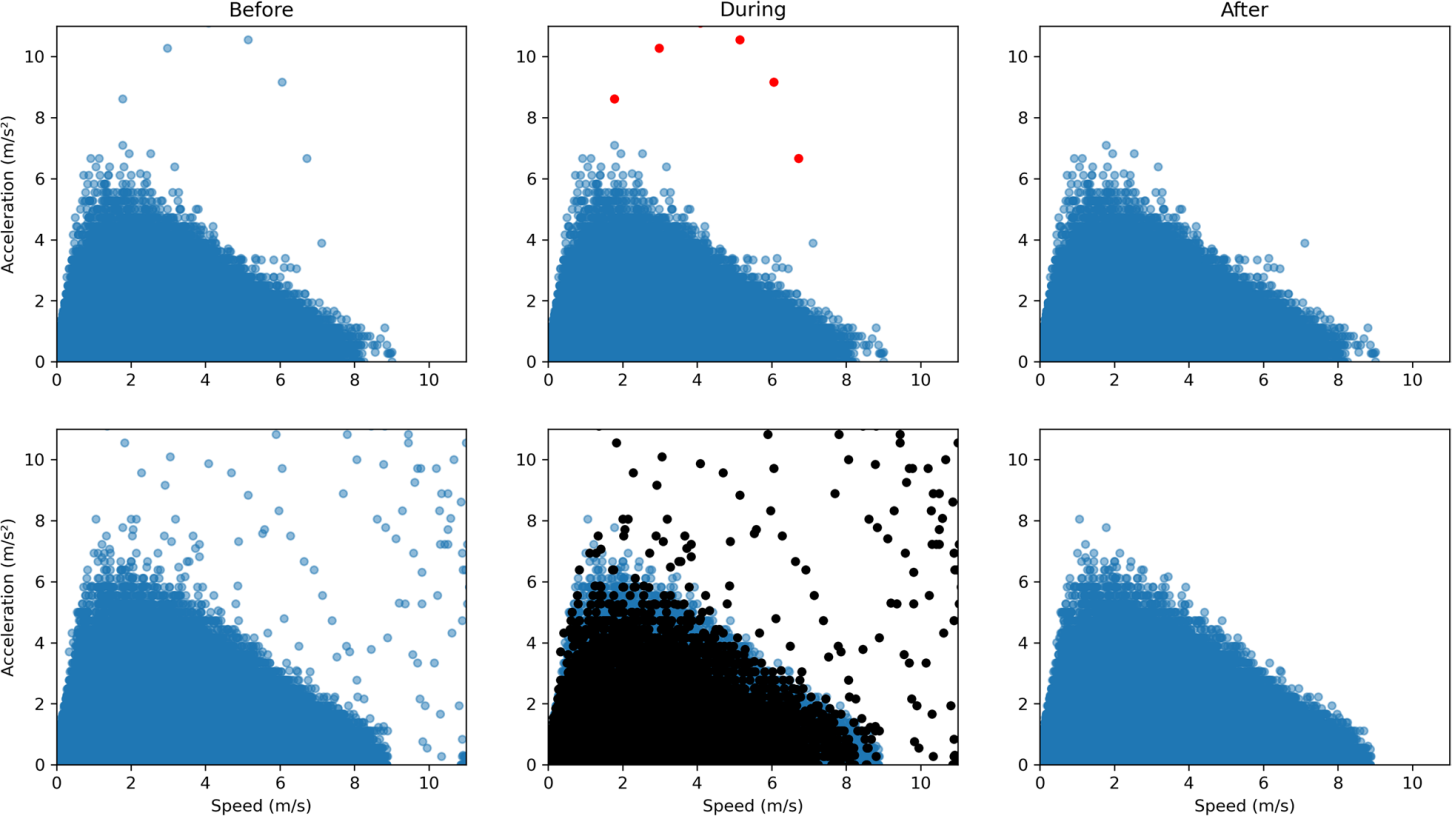
Example of outliers’ identification in two individual GPS-induced *AS* relationships (computed from all training sessions and official games; one row for each player). From left to right: before, during and after outliers’ identification. The red dots are measurement errors corrected with density-based clustering algorithm (DBSCAN) whereas the black dots represent values corrected by 3σ-rule

Before considering the relationship between maximal acceleration and maximal speed [19], outlier points (*i.e.*, artefacts or measurement errors) from GPS data were identified according to the distribution of theoretical maximal velocity (μ_V_, σ_V_) and theoretical maximal force (μ_F_, σ_F_), considered as norms in rugby union [27]. By applying the 3σ-rule [29], an acceleration greater than μ_F_ + 3σ_F_ or a velocity greater than μ_V_ + 3σ_V_ were considered unlikely. The force–velocity relationship being close to a linear relation, any value above the line formed by the y-intercept of μ_F_ + 3σ_F_ and the x-intercept of μ_V_ + 3σ_V_ were also considered unlikely and therefore discarded. These values were counted for each session. Above 15 unlikely values (corresponding to 1.5 s of consecutive improbable values), all player’s data was dropped out for that session (Fig. 1; black points). Except for measurement errors inherent to the GPS (n = 14; deleted from the dataset), only a single case with two defective days, and 8 cases with one defective day were identified. This is particularly important as a Gaussian filter was used afterwards [19] (in that case, a single outlier can affect all its neighboring values). By applying the density-based clustering algorithm (DBSCAN) [30], values without neighbors in the AS area were identified as outliers (Fig. 1; red dots). This method allows to identify the values far enough from each other’s. Physically, two consecutive values in time from a trajectory are close in space. Thus, a value without neighbor in the *AS* space could be considered as an outlier. Briefly, DBSCAN algorithm needs (i) the number of values in a neighborhood to consider a dot as a core value, and (ii) the size of the neighborhood which corresponds to the maximum distance between two values quantified as the Euclidean distance [$Dist\left({P}_{t}, {P}_{t+ \Delta t}\right)$] between two consecutive values ${P}_{t}$ [defined by *acceleration* as $a\left(t\right)$ and speed as $s\left(t\right)$] and ${P}_{t+ \Delta t}$ with $\Delta t$ the associated time difference. This $\Delta t$ corresponds to the sensor sampling frequency (10 Hz).

Therefore, by definition of the Euclidean distance:

1 $$\begin{array}{*{20}c} {Dist\left( {P_{t} , P_{t + \Delta t} } \right) = \sqrt {\Delta a^{2} + \Delta s^{2} } } \\ \end{array}$$

With $\Delta s$ (resp. $\Delta a$) the speed difference between two consecutive speeds (resp. accelerations):

2 $$\begin{gathered} \Delta s = s\left( {t + \Delta t} \right) - s\left( t \right) \hfill \\ \Delta a = a\left( {t + \Delta t} \right) - a\left( t \right) \hfill \\ \end{gathered}$$

The classic approach used by Morin et al. [11] to quantify a sprint is to define $s\left(t\right)$ as exponential over time:

3 $$\begin{array}{*{20}c} {s\left( t \right) = S_{\max } \left( {1 - e^{{ - \frac{t}{\tau }}} } \right) } \\ \end{array}$$

With ${S}_{{\text{max}}}$ the maximal speed (in m s^−1^) reached at the end of the acceleration and τ the acceleration time constant (in s).

By deriving (3) over time:

4 $$\begin{gathered} a\left( t \right)\frac{ds}{{dt}} \hfill \\ a\left( t \right) = \frac{1}{\tau }s\left( t \right) + \frac{1}{\tau } \hfill \\ \end{gathered}$$

From (2) and (4):

5 $$\begin{array}{*{20}c} {\Delta a = \frac{1}{\tau } \Delta s} \\ \end{array}$$

By factoring from (1) and (5):

6 $$\begin{array}{*{20}c} {Dist\left( {P_{t} , P_{t + \Delta t} } \right) = \left| {\Delta s} \right|\sqrt {1 + \frac{1}{{\tau^{2} }}} } \\ \end{array}$$

From (2) and (3):

7 $$\begin{array}{*{20}c} {\Delta s = S_{\max } e^{{ - \frac{t}{\tau }}} \left( {1 - e^{{ - \frac{\Delta t}{\tau }}} } \right) } \\ \end{array}$$

From (3) and (7):

8 $$\begin{array}{*{20}c} {\Delta s = \left( {S_{\max } - s\left( t \right)} \right)\left( {1 - e^{{ - \frac{\Delta t}{\tau }}} } \right)} \\ \end{array}$$

rom (6) and (8):

9 $$\begin{array}{*{20}c} {Dist\left( {P_{t} , P_{t + \Delta t} } \right) = \left| {S_{\max } - s\left( t \right)} \right|\left| {1 - e^{{ - \frac{\Delta t}{\tau }}} } \right|\sqrt {1 + \frac{1}{{\tau^{2} }} } } \\ \end{array}$$

Finally:

10 $$Dist\left( {P_{t} , P_{t + \Delta t} } \right) \le \left| {S_{\max } } \right|\left| {1 - e^{{ - \frac{\Delta t}{\tau }}} } \right|\sqrt {1 + \frac{1}{{\tau^{2} }} }$$

With $\tau = 1.19\;{\text{s}}$, $S_{\max } = 9.89\;{\text{m}}\;{\text{s}}^{ - 1}$ from Morin et al. [11] and $\Delta t = 10\;{\text{Hz}} = 0.1\;{\text{s}}^{ - 1}$:

$$Dist\left( {P_{t} , P_{t + \Delta t} } \right) \le 1.13$$

Thus, the parameters for the DBSCAN algorithm are 1.13 for the maximum distance between two samples and 3 for the number of samples in a neighborhood (i.e., ${P}_{t}$, its previous one, ${P}_{t- \Delta t}$, and its next one, ${P}_{t+ \Delta t}$).

### Data Processing

**Fig. 2:**
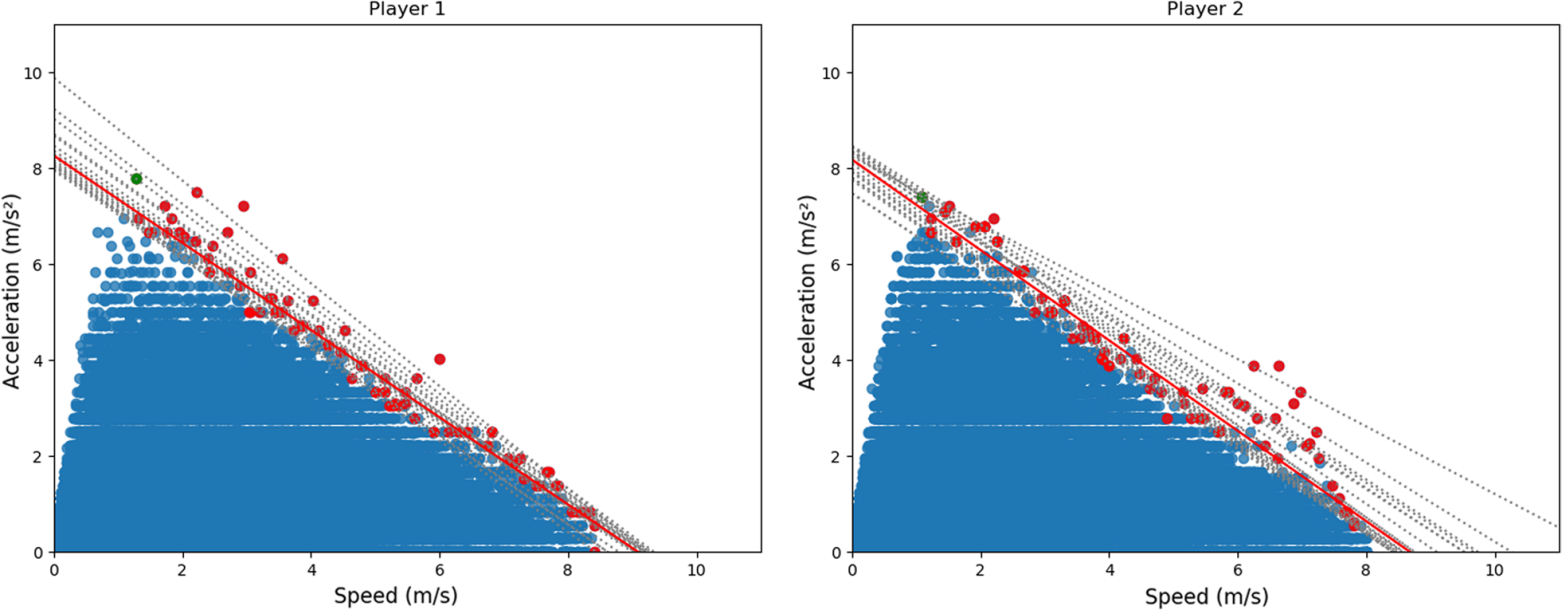
Example of quantile regressions for selected dots, left and right panels represent a high variance in *A*_*O*_ and in *S*_*O*_, respectively

After removal of outliers, the *AS* linear relationship was modeled for each player. Then, the maximal acceleration was detected (Fig. 2; green dots). From this maximal acceleration to the individual maximal speed reached, two values at maximum acceleration for every 0.2 m.s^−1^ sub-interval were selected (Fig. 2; red dots) to generate a linear *AS* profile. In some cases, an unequal variance in *A*_*0*_ (Fig. 2, left panel) or in *S*_*0*_ (Fig. 2, right panel) was observed, resulting in higher uncertainty on *A*_*0*_ and *S*_*0*_ values.

Considering the Mean Square Error (MSE) as the cost function to be minimized, the furthest values from the linear regression have a very strong weight in the regression calculation. Meanwhile, the values located far below the linear regression are not necessarily the most relevant. Changing the cost function by ${L}_{\gamma }$, as defined in (11), yields a quantile regression with several advantages.

11 $${L}_{\gamma }\left(y, {y}_{p}\right)= \sum_{{y}_{i}<{y}_{p, i} }\left(1-\gamma \right) \left|{y}_{i}-{y}_{p,i}\right|+\sum_{{y}_{i}\ge {y}_{p, i} }\left(\gamma \right) \left|{y}_{i}-{y}_{p,i}\right|$$

With $y$ observed values, ${y}_{p}$ predicted values and $\gamma \in \left]\mathrm{0,1}\right[$.

The first advantage is the change of $({y}_{i}-{y}_{i}^{p})$ ^2^ in the MSE quadratic term with $\left|{y}_{i}-{y}_{i}^{p}\right|$. This change places less weight to values far from the predicted linear regression. The second advantage is the contribution of the $\gamma$ term. When $\gamma$ < 0.5, a higher weight to the values under the obtained linear regression is given. If $\gamma$ > 0.5, the linear regression is boosted by a higher weight. By varying $\gamma$ between 0.05 and 0.95, more or less weight is given to the values. The variation of $\gamma$ produced different straight linear regressions and thus an interval of possible values for* A*_*0*_, *S*_*0*_ and for the slope, *i.e.*, overall orientation of the *AS* profile (computed as *AS*_slope_ =—*A*_0_ / *S*_0_) (Fig. 2; linear regressions). Thus, for each player and sessions, different *A*_*0*_, *S*_*0*_ and *AS*_slope_ values are obtained. Their averaged values provide information on the most relevant *A*_*0*_ and *S*_*0*_ values while the standard deviation of this multiple linear regressions indicates the accuracy of the method. This new method estimating the *AS* profile provides an error of measurement around *A*_*0*_, *S*_*0*_ and *AS*_slope_. The source code is available on a GitHub page: https://github.com/NthnMgns/acceleration-speed-profiling.

### Statistical Analysis

In order to assess the reliability of the *AS* profile within the collection period, data were compared over two 2-weeks phases as described elsewhere [19]. Briefly, the inter-phase reliability for *A*_*0*_, *S*_*0*_ and *AS*_slope_ was quantified through the change in the mean (systematic error), the standard error of measurement (SEM, random error), both expressed in raw units and in percentage of mean values, and the inter-class correlation coefficient (ICC) between two 2-weeks phases datasets. *A* and *S* were compared between positions and session types using ANOVA and Tukey post hoc tests when the significant threshold had been reached. The level of significance was set at p = 0.05.

## Results

All *AS* individual profiles showed linear relationships with high coefficient of determination (all r^2^ > 0.81).

### Inter-Phase Reliability

Percentages of standard error measurement (SEM) fluctuate between 4.2 and 8.6%, with an* S*_*0*_, *A*_*0*_ and *AS*_*slope*_ Inter-class Correlation Coefficient (ICC) between the two phases of 0.92, 0.72 and 0.73, respectively (Table 1).

**Table 1:** Main variables of the individual acceleration-speed (*AS*) profile for the two training phases analyzed

Variable	Phase 1	Phase 2	Raw difference (Phase 2–Phase 1)	Raw difference (% from Phase 1)	SEM (raw units)	SEM (%)	ICC
*S*_*0*_ (m s^−1^)	8.29 ± 0.91	8.34 ± 0.83	0.36 ± 0.30	4.38	0.35	4.18	0.92
*A*_*0*_ (m s^−2^)	7.25 ± 0.56	7.30 ± 0.60	0.43 ± 0.34	5.87	0.37	5.13	0.72
*AS*_*slope*_ (s^−1^)	− 0.88 ± 0.12	− 0.88 ± 0.09	0.08 ± 0.06	− 8.51	0.08	8.59	0.73

*A*_*0*_: maximal theoretical acceleration; *S*_*0*_: maximal theoretical speed; *AS*_slope_: slope of the *AS* profile; SEM: standard error of measurement; *ICC* inter-class correlation coefficient

### AS Profile Among Positions

*A*_*0*_, *S*_*0*_ and *AS*_slope_ according to positions are presented in Table 2. Centre players showed significant superior mean values of 8.40 ± 0.06 m.s^−2^ and 9.71 ± 0.60 m.s^−1^ for *A*_0_ and *S*_0_, respectively, than their second row counterparts (7.65 ± 0.48 m.s^−2^ and 7.82 ± 0.53 m.s^−1^ for *A*_0_ and *S*_0_, respectively) (Table 2).

**Table 2:** mean and error of measurement of theoretical maximal acceleration (y-intercept of the *AS* linear relationship; *A*_*0*_), theoretical maximal running speed (x-intercept of the *AS* relationship; *S*_*0*_) and slope of the *AS* profile (slope of the *AS* relationship; *AS*_slope_) by positions

	*A*_*0*_ (m.s^−2^)	*S*_0_ (m.s^−1^)	*AS*_slope_ (s^−1^)	Significant differences of *A*_0_ *vs*	Significant differences of *S*_0_ *vs*
First row (n = 10)	7.86 ± 0.41	7.68 ± 0.62	− 0.98 ± 0.17		Third row, Half scrum, Half back, Centre, Wing and Back
Second row (n = 7)	7.65 ± 0.48	7.82 ± 0.53	− 0.98 ± 0.11	Centre	Third row, Half scrum, Half back, Centre, Wing and Back
Third row (n = 10)	8.08 ± 0.90	8.94 ± 0.50	− 0.91 ± 0.12		First row, Second row and Centre
Half scrum (n = 3)	8.28 ± 0.06	9.18 ± 0.06	− 0.90 ± 0.01		First row and Second row
Half back (n = 2)	7.58 ± 1.18	9.28 ± 0.33	− 0.82 ± 0.16		First row and Second row
Centre (n = 3)	8.40 ± 0.06	9.71 ± 0.60	− 0.87 ± 0.08	Second row	First row, Second row and Third row
Wing (n = 7)	8.00 ± 0.58	9.30 ± 0.37	− 0.86 ± 0.06		First row and Second row
Back (n = 3)	8.44 ± 0.76	9.42 ± 0.14	− 0.90 ± 0.08		First row and Second row

**Fig. 3:**
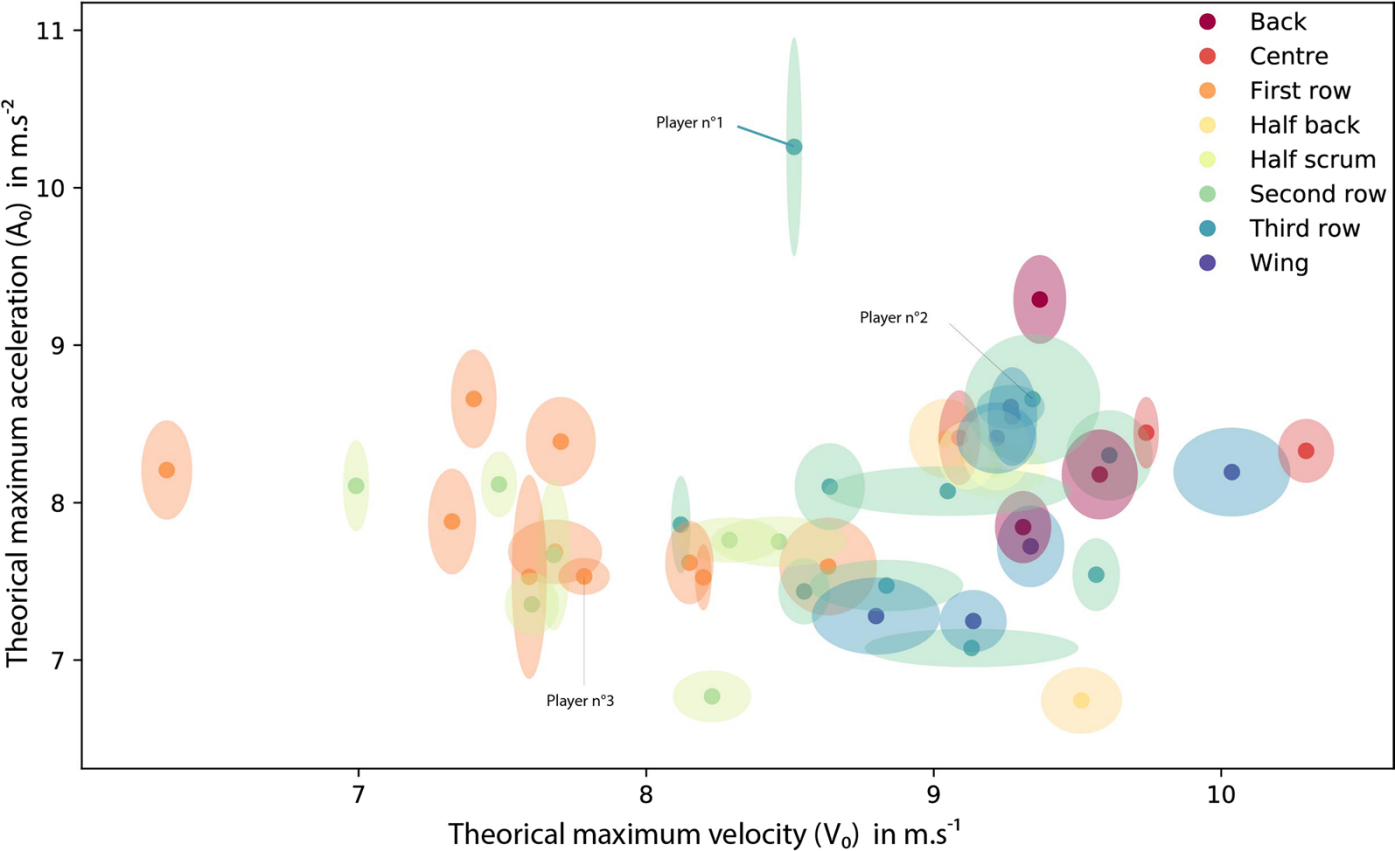
Mean and error measurement (provided by the quantile regression method) of theoretical maximal acceleration (y-intercept of the AS linear relationship; *A*_0_) and theoretical maximal running speed (x-intercept of the AS relationship; *S*_0_*)* for each rugby union player. Positions are represented by color

Computed mean and error measurements (with its x and y error measurement expressed by the size of the area) for *A*_*0*_ and *S*_*0*_ are displayed in Fig. 3 for each player and position. Three different *AS* profiles are presented independently of position: (i) a high mean *A*_*0*_ with a large error measurement (10.26 ± 1.39 m.s^−2^) with an averaged *S*_0_ with a low error measurement (8.51 ± 0.05 m.s^−1^) (Fig. 3; Player n°1), (ii) large error measurement on both *A*_0_ (8.66 ± 0.83 m.s^−2^) and *S*_0_ (9.34 ± 0.47 m.s^−1^) (Fig. 3; Player n°2), and (iii) low error measurement on both *A*_0_ (7.53 ± 0.23 m.s^−2^) and *S*_0_ (7.78 ± 0.1 m s^−1^) (Fig. 3; Player n°3).

### AS Profile Among Different Trainings and Games

**Fig. 4:**
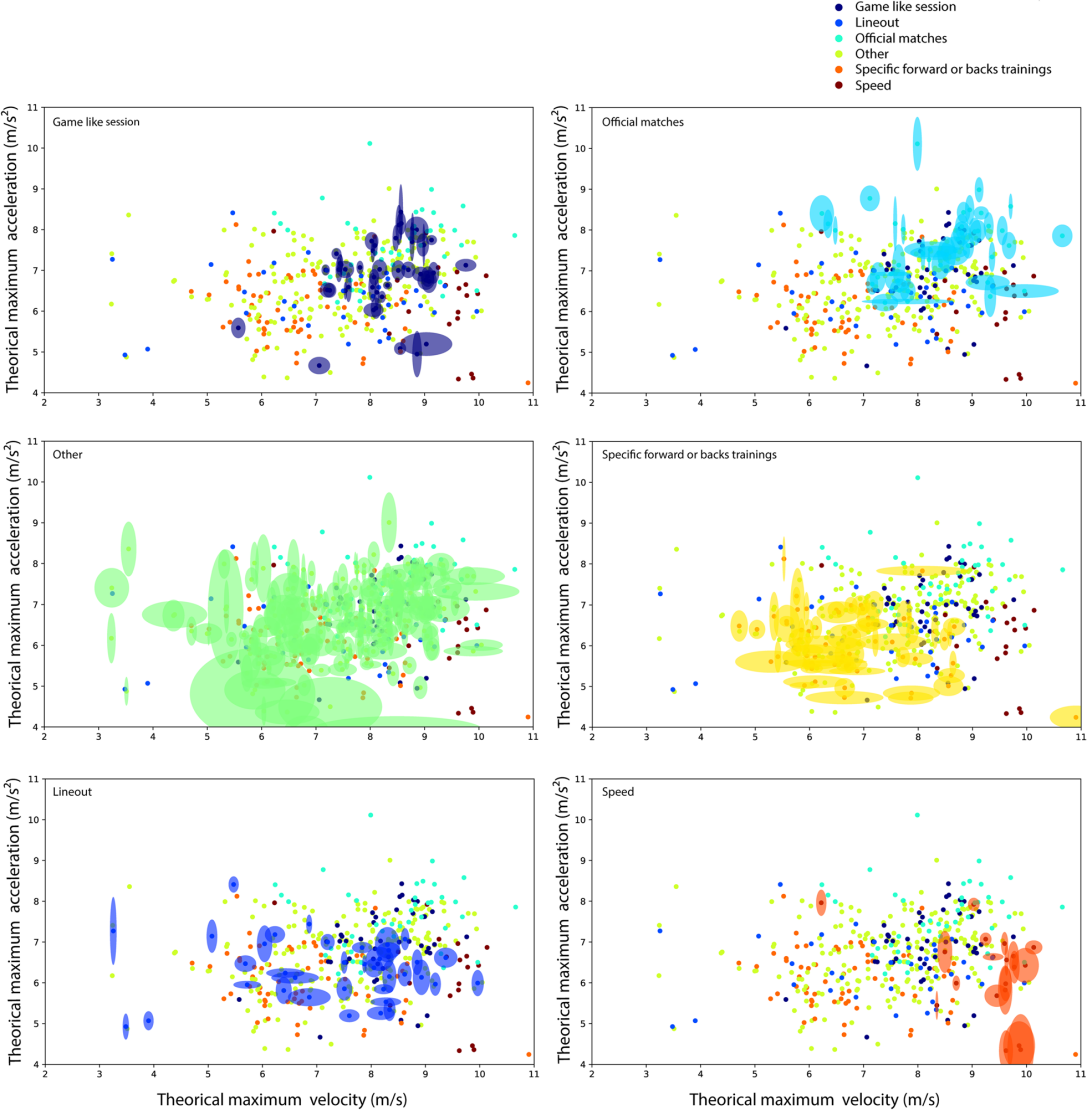
Mean and error measurement (provided by the quantile regression method) of theoretical maximal acceleration (y-intercept of the AS linear relationship; A0) and theoretical maximal running speed (x-intercept of the AS relationship; S0) for each rugby player. Types of training and game are represented by different color

Computed mean and error measurements for *A*_*0*_*, S*_*0*_ and *AS*_*slope*_ for each rugby training session and game are detailed in Table 3. Mean *A*_*0*_ calculated from official games was significantly higher than from all others trainings (Table 3). Mean *S*_*0*_ calculated from speed sessions was significantly higher than from all others trainings and games (Table 3). Computed mean and error measurements for each player for *A*_*0*_ and *S*_*0*_ are displayed in Fig. 4 on training or game sessions (with the x and y magnitude of error measurement expressed by the size of the area). Mean and error measurements ranged from 6.08 ± 0.75 m.s^−2^ (specific forward or back trainings) to 7.71 ± 1.05 m.s^−2^ (official game) for *A*_*0*_ and from 6.79 ± 1.13 m.s^−1^ (Specific forward or back trainings) to 9.28 ± 0.88 m.s^−1^ (speed sessions) for *S*_*0*_ (Table 3). Large differences on error measurement were observed between the type of session (Fig. 4). Scrimmage sessions and speed sessions appeared with the lowest SEM on *S*_*0*_ (0. 77 and 0.88 m.s^−1^, respectively; Table 3); yet inversely, other sessions (lineout and other sessions) presented the highest error measurement on *S*_*0*_ (1.63 and 1.30 m.s^−1^, respectively; Table 3 and Fig. 3).

**Table 3:** Mean and error measurement of theoretical maximal acceleration (y-intercept of the *AS* linear relationship; *A*_0_) and theoretical maximal running speed (x-intercept of the *AS* relationship; *S*_0_) by type of rugby sessions

	*A*_*0*_ (m s^−2^)	*S*_*0*_ (m.s^−1^)	*AS*_slope_ (s^−1^)	Significant differences of *A*_0_ *vs*	Significant differences of *S*_0_ *vs*
Official games	7.71 ± 1.05	8.39 ± 0.99	− 0.93 ± 0.07	#, $, †, £, ¥	†, £, ¥
Speed sessions	6.22 ± 1.04	9.28 ± 0.88	− 0.68 ± 0.07	*	$, †, £, ¥
Scrimmage sessions	6.90 ± 0.86	8.22 ± 0.77	− 0.84 ± 0.05	*, †, £	#, †, £, ¥
Specific forward or back trainings	6.08 ± 0.75	6.79 ± 1.13	− 0.93 ± 0.10	*, #, ¥	*, #, $, ¥
Lineout	6.32 ± 0.74	7.25 ± 1.63	− 0.92 ± 0.09	*, $	*, #, $
Others	6.66 ± 0.89	7.55 ± 1.30	− 0.91 ± 0.09	*, †	*, #, $, †

*Significantly different *vs*. Official games, # significantly different *vs*. Speed sessions, $ significantly different *vs*. scrimmage sessions, † significantly different *vs*. Specific forward or back trainings, £ significantly different *vs*. Lineout, ¥ significantly different *vs*. Others

## Discussion

This study aimed to automatize the individual GPS-derived *in-situ AS* profiling capable of generating an error of measurement on *A*_*0*_ and *S*_*0*_ components in reference to training type, games and positions in male rugby union players. According to soccer-related studies [19, 21], this study confirms that individual *in-situ AS* profiles can be computed from rugby union GPS data and provide positional benchmarks for both training and competitive scenarios. Furthermore, two major improvements including the automatic detection of atypical data during processing and the computation of an error measurement on the *A*_*0*_ and *S*_*0*_ components for each training sessions and games may enable the daily integration of *AS* profiles in players’ monitoring.

### An automatized and Refined Method

All *AS* individual profiles show linear relationships with high r^2^, similarly (albeit slightly lower) to those measured in previous studies [19, 20]. This difference is possibly due to the fact that, in the present study, several linear regressions were carried out without shrinkage (instead of a single one with removal of the points too far from the linear regression) to calculate an error of measurement.

The very good reliability for *AS* profiles determined two weeks apart (all standard error of measurements < 8.6%, Table 1) is in accordance with *in-situ AS* profile in soccer [19, 20], and in line with standardized force–velocity sprint testing [6, 31]. Nevertheless, the residual variance may be contingent upon factors such as the quality of the sampled GPS signal, environmental and tactical conditions during both trainings and matches, training specificity (*e.g.*, speed session *vs*. specific forwards training), and inherent hardware and software characteristics among other considerations.

*AS* profiles can change over a professional rugby union season [32] and are therefore likely to influence the inter-phase systematic differences observed in the present dataset. For example, in elite youth soccer players, the number and content of sessions affect *S*_*0*_ [20]*.* High-speed exposure seems to be essential to build a reliable *in-situ AS* profile [20]*.* Here, the proposed method makes it possible to independently identify the number of points or the degree of high-speed exposure the level of uncertainty on the *A*_0_ *and* S_0_ components. This refinement constitutes a step forward for a daily use of ecological data by considering data collection and method reliabilities.

### AS Profiles Sensibility to Positions, Training Typologies and Games

The increasing use of field testing questions the *in-situ AS* measurement reliability [33]. One approach to assess the reliability of a new measure is to compare it to the gold standard and/or evaluate its capability to differentiate players by position or level. The automatized method developed here is able to detect different range of *A*_*0*_ and *S*_*0*_, reflecting the major inter-player differences in *AS* capacities, even in a highly-trained population, in accordance with previous method [19, 20]. Indeed, we find similar position-specific *A*_*0*_ and *V*_*0*_ values in reference to single straight sprinting test (*i.e.*, 30-m performance with radar and dual-beam infrared timing gates), which have been carried out on rugby union players [27]. Recently, Glaise et al. [34] reports similar components of the sprint force–velocity profile according to positional group (forwards *vs*. backs). These parameters of *AS* profile also depend on the players’ position. Indeed, backs produce higher *S*_*0*_ than forwards in accordance with the game demands and inherent capabilities. These players are faster over several distance ranging from 20 to 100 m [2], involved in more sprints and in larger sprinting distance, and completed more high-speed running compared to the forwards [35]. Interestingly, this automatized method was also tested to identify differences of *A*_*0*_ and *S*_*0*_ components by type of session. Highest *A*_*0*_ are obtained during games, while greatest *S*_*0*_ are attained during games and speed sessions. These values are in line with the competitive demands of a match, where players typically sprint within the range of 10–20 m [36]. The elevated *S*_*0*_ observed during speed session could also result from the greater distance available for sprinting. The achievement of *S*_*0*_* or V*_*max*_*,* prerequisite to reliably build the AS profile, suggest that rugby players should be tested over distances greater than 40 m [36]. This circumstance may be more frequently encountered during training sessions compared to actual game situations. One possible explanation lies in the stochastic and intermittent nature of rugby union that require a great number of accelerations regardless of position during dueling, tackling, rucking, acting as a support player, or running decoy lines to distract the opposition, or covering in defense. The fact that the highest *S*_*0*_ (9.28 ± 0.88 m.s^−1^) are measured during the speed sessions can be explained by the need to achieve higher percentages of maximum speed (*i.e.*, ≥ 95%) [20]. The present study is the first to consider *AS* profiling in reference to training and games. Waiting for further studies to deepen our understanding of training- and game-induced responses on *AS* components, the present findings are in line with the proof-of-concept developed in professional soccer players [19, 20] and open an era for regular monitoring of *AS* profiles and individualization of training programs, that may be easy to implement (without specific testing apparatus required, but only GPS data that may be passively collected *in-situ*).

### Competitive Reserve

This study reveals that the highest *A*_*0*_ are obtained during games, while greatest *S*_*0*_ are attained during games and speed sessions. This is why it is essential to measure these competitive parameters which represent the reference under maximal competitive constraints. The comparison obtained during other types of training or game can provide an understanding of the physiological strain of each session and express a "competitive reserve". This "competitive reserve" illustrates what we can hope to see put into play in the next game or what certain players who know how to manage themselves can dig deep within themselves to implement it at the appropriate time (money time, in the opposing 22 m, or during key moment of the game). This "competitive reserve" can also illustrate the load margin to reach *A*_*0*_ or *S*_*0*_ during trainings. This can be considered as the benchmark value for individual standardization. The 100% represents the individual competitive demand, and each exercise is a distance to this maximum. Each quantifiable parameter of training can thus be assessed in relation to the most demanding competitive scenarios. A characterization of each sessions performed based on the distance from the competitive demands can thus be provided. Therefore, it becomes feasible to position the player between two competitions in terms of his capacity to recover maximum efficiency or his kinetics to return to peak performance.

### Practical Application

To the best of our knowledge, this study is the first to provide an automatized detection of *AS* time series atypicity, able to generate individual *in-situ AS* profiles in professional rugby union. This method facilitates data processing and identification of outliers, allowing for the provision of error measurements on the *A*_*0*_ and *S*_*0*_ parameters. Compared to the single straight sprinting test-based force–velocity profiling, generating *in-situ AS* profiles allows to contextualize performance data (*e.g.*, measuring acceleration and speed in ecological settings), representative of rugby union specificities or constraints [37]. Of relevance, the method developed in the present study provides an error measurement for both *A*_*0*_ and *S*_*0*_ components that may allow to monitor the inter-day variation in each individual *AS* profile for rugby union players’ follow-up [38], and data-driven training decisions [39, 40]. In our view, collecting continuous data over time would permit to understand changes in individual *AS* profile in reference to a specific training or strength and conditioning intervention (*e.g.*, force- or acceleration- *vs*. speed-based training or generic [running-based high-intensity intermittent training] *vs*. sport-specific [small-sided games] training) or over time (*e.g.*, dose–response relationship) or over different period of the season (*e.g.*, regular season *vs*. play-off). Also, according to individual *AS* profile, specific personalized work to improve acceleration and sprint capacities can be proposed with daily monitoring to control the training quality or fatigability status, thereby allowing to avoid overstress and injury. Third, “top-up” sessions could be implemented if the desired *A*_*0*_ and *V*_*0*_ are not achieved during targeted training sessions. Such daily information could be considered as a predictor of a readiness score, which may have usefulness in the understanding of kinetics of return to individual *A*_*0*_ and *S*_*0*_ post-game, between games or to follow-up the recovery of injuries or their prevention [19] (return-to-sport or return-to-performance processes). Overall, this method may be extremely convenient for an on-field use by sport practitioners due to its time-saving advantage and supplemental information (*i.e.*, error measurements) on the reliability of the *AS* profile. This makes such an automatized profiling key to the performance management process (talent identification, training monitoring and individualization, return-to-sport).

### Limitations and Perspectives

The determination of individual *in-situ AS* profiles is primarily dependent on data quality [19]. In this study, acceleration was computed by deriving speed from GPS raw data, and not from the accelerometer embedded in the GPS sensor. The reason was to avoid any shock, impact, tackle, changes of direction, and all other sudden forces susceptible to alter the vectorial acceleration (Newton's 2nd law) which depends on the variation of speed rather than forces, for a given time. Future development would permit to directly use data from accelerometer instead of extrapolating acceleration from GPS-based data. In this view, providing more weight for the greatest acceleration at a given speed (*i.e.*, the moment when the athlete provides a maximum effort) would be relevant to modify the ${\varvec{\gamma}}$ value.

## Conclusion

This study provides an automatized method to generate individual *in-situ* acceleration-speed profiles derived from GPS data in professional rugby union players. It has helpful refinements such as an automatic detection of atypical data and the computation of error measurement of theoretical maximal acceleration and speed components for each position, training session and game. Available through open-source repositories, such an easy-to-implement approach may facilitate its use for testing, training monitoring, talent identification, injury prevention or return-to-sport.

## Data Availability

Dataset are unavailable due to sensitive details pertaining to the club's operations. For the purpose of open research, a data sharing process involving anonymized data under a different research protocol can be established by contacting the corresponding author.

## Abbreviations

AS: Acceleration speed
GPS: Global positioning system
*A*_0_: Maximal theoretical acceleration
*S*_0_: Maximal theoretical speed
*V*_0_^2^: Maximal theoretical velocity
*AS*_slope_: Acceleration-speed slope
LPS: Local positioning systems
CNIL: Commission Nationale de l’Informatique et des Libertés
ProD2: Professional division 2
DBSCAN: Density-based clustering algorithm
SEM: Standard error of measurement
ICC: Inter-class correlation coefficient
ANOVA: Analysis of variance

## Code Availability

The source code can be found on our GitHub page: https://github.com/NthnMgns/acceleration-speed-profiling.
